# Burnout among medical doctors working in paediatric intensive care units in Bulgaria

**DOI:** 10.3389/fped.2026.1791485

**Published:** 2026-06-01

**Authors:** Mergyul Halilova, Darina Krumova, Tanya Zlateva, Tanya Teneva, Dimitar Pechilkov, Bogdan Mladenov, Blagomir Zdravkov, Ivan Ivanov, Daniela Avdjieva, Yoto Yotov, Violeta Iotova

**Affiliations:** 1 Department of Paediatrics, Medical University of Varna, Varna, Bulgaria; 2 Department of Paediatrics, UMHAT “Sveta Marina”, Varna, Bulgaria; 3 Department of Paediatrics, Medical University of Sofia, Sofia, Bulgaria; 4 Department of Paediatrics, The Specialized Children's Hospital “Prof. Dr. Ivan Mitev”, Sofia, Bulgaria; 5 Paediatric Postoperative Intensive Care Unit and Reanimation, National Cardiologic Hospital, Sofia, Bulgaria; 6 Paediatric Anesthesiology and Intensive Care Unit, UMHATEM “N.I.Pirogov”, Sofia, Bulgaria; 7 Department of Paediatrics and Medical Genetics, Medical University of Plovdiv, Plovdiv, Bulgaria; 8 First Depatrtment of Internal Medicine, Medical University of Varna, Varna, Bulgaria

**Keywords:** Bulgaria, burnout, healthcare providers, paediatric intensive care unit, survey

## Abstract

**Introduction:**

Paediatric intensive care units (PICUs) provide special and comprehensive care and treatment to critically ill children. These factors lead to the exposure of the personnel to stress and the risk of developing burnout syndrome. The aim of the current study was to evaluate burnout syndrome in PICUs’ higher medical staff.

**Methods:**

and participants: In order to evaluate burnout syndrome among physicians employed at PICUs, we carried out our study between September and December of 2023. With the authors’ consent, we used a survey that was published in June 2023 in Acta Paediatrica.The survey was distributed to all physicians working in the existing PICUs in Bulgaria after being translated and reviewed.

**Results:**

A total of 37/43 (86%) doctors and two clinical psychologists completed the survey. More women than men took part in the study. Of the respondents, 70.2% were paediatricians, trainees in paediatrics or had a pediatric subspecialty, and 29.7% were specialists in Anesthesiology and Intensive Care. In total, 70.3% of the staff reported working between 40 and 50 h/week, and 24.3% working more than 50 h/week; 73% of the employees experienced an episode of overheating, depression, and anxiety. Of all, 75.6% felt exhausted from work, and 40.5% defined themselves as “crushed”. Despite the difficulties that the staff faced, the majority of the doctors express satisfaction with their work and would choose the same specialty again.

**Conclusion:**

This study presents for the first time quantitative data on Bulgarian physicians’ burnout in PICUs. Burnout among healthcare professionals is a worldwide issue that has a detrimental effect on staff productivity, patient care quality, and hiring medical personnel. Specific solutions are required, such as the establishment of paediatric intensive care as a distinct specialty.

## Introduction

The Paediatric Intensive Care Unit (PICU) is a hospital unit that provides diagnosis and treatment of children from 0 to 18 years of age with critical, life-threatening illnesses (1). The most seriously ill children and their families receive all-encompassing care from the personnel at PICUs (2). The goal of critical care is to maintain a child's quality of life as much as possible, in addition to preserving their life (3). Due to the high level of empathy that is required to care for children at PICUs and the complexity of the relationships with their families, working in these units is very specific (4). Split-hour work schedules, ethical dilemmas like incapacity or death, unclear prognoses such as severe congenital anomalies compound the work. Additional features are the highly technical nature of the provided care, the daily exposure to the physical and psychological suffering of these children and their families, and the exposure to some extraordinary and unpredictable situations (5). Everything mentioned above has an emotional and professional impact on the PICU staff, often even without the fact being realized by the practitioners.

These complex factors lead to the exposure of the personnel working in PICUs to the risk of developing pathological conditions such as burnout syndrome or depression. Burnout syndrome was first described by Herbert Freudenbenger in 1970s as a state of emotional exhaustion, reduced physical and physiological energy and decrease motivation at work. It is commonly conceptualized as a multidimensional construct consisting of three components: Emotional Exhaustion, Depersonalization, and reduced Personal Accomplishment. These three dimensions of burnout are thought to function in a continuum. “Exhaustion” usually develops first, in response to overload, being followed by negative reactions such as detachment – “depersonalization”. If these continue, severe repercussions to the life of the individual may occur, resulting in “diminished accomplishments” (6). Among the available instruments for assessing burnout, the Maslach Burnout Inventory (MBI), developed by Susan E. Jackson and Christina Maslach, is the most widely used and validated tool, particularly in healthcare settings. The MBI – Human Services Survey (MBI-HSS) consists of 22 items distributed across three subscales: Emotional Exhaustion, Depersonalization, and Personal Accomplishment. Each item is rated on a Likert scale, with higher scores in Emotional Exhaustion and Depersonalization and lower scores in Personal Accomplishment indicating higher levels of burnout (7). Furthermore, recent research has demonstrated that staff members in intensive care units also report high levels of work-related post-traumatic stress disorder (8–10). Physicians of all specialties from Europe, Australia, and USA report that the main factor contributing to burnout is the repetition of night work in the form of in-house duty, with the total number of hours worked per week being a risk factor for severe burnout (11, 12). Critical care staff members are at an increased risk of psychological problems due to constant exposure to grief, trauma, and death. Those who work with children are particularly vulnerable (13–15). Situations in which quick and critical decisions are made without taking into account their accuracy or the consequences are not to be ignored. They affect the physician who is directly responsible for the consequences both emotionally and mentally. Nevertheless, fewer studies have looked at the possible risk or protective factors linked to burnout, and even fewer have looked at the prevalence of burnout among PICU staff (15). Given that burnout can negatively impact a physician's physical and mental well-being as well as their sense of self in the workplace, it is evident that high levels of burnout among health professionals have significant clinical and health effects on the system. This in turn could have a detrimental effect on staff recruitment and retention as well as on the quality, safety, and satisfaction of patient care (16–18).

Bulgaria does not currently have a distinct specialty for working in PICUs or a specific status for employees as has been implemented in the majority of countries. Due to the high cost of PICU services, public medical institutions often lack sufficient resources to invest in the physical and emotional well-being of their personnel. Despite the urgent need to inform hospital management and the broader healthcare community, burnout among PICU staff in Bulgaria has not yet been systematically investigated.

The aim of the current study was to evaluate the signs of burnout syndrome among PICUs' higher medical staff.

## Materials and methods

### Study design

We performed a survey among health personnel in PICU between 15 September and 15 December 2023 using the methodology of a similar study in France, (https://onlinelibrary.wiley.com DOI: 10.1111/apa.16871). After permission from the authors was obtained, we translated the survey in Bulgarian and adaptated the terms to the local working conditions. Both translation and adaptation were carried out by a physician with more than 5 years experience at PICU and revised blindly by a supervisor. The language adaptations concerned mainly terms of the working environment and personnel structure. We followed the Cross-Cultural Survey Guidelines of the University of Michigan (https://ccsg.isr.umich.edu/chapters/adaptation/0). The survey contained 93 questions and was first made available online through the Google Forms platform (Supplementary Material).

### Survey content

The anonymous questionnaire included questions from the following groups:

- gender, age, and status of the medical facility;
- specialty(ies) of the respondent;
- working hours, number of shifts, workload;
- recognition and discrimination at the workplace;
- emotional stress related to work, family situation, non-professional activities;
- monthly remuneration and overall professional satisfaction.

### Data collection

The questionaire was distributed to the heads of the five existing PICUs in Bulgaria, all affiliated to University hospitals for review and comments. Once the final version of the survey was completed and approved, it was made available to the survey respondents who were all fully employed higher medical personnel at the units. Since Paediatric intensive care is not a recognized specialty in Bulgaria, physicians from various specialties may work in these wards. Temporary residents were not invited to the study.

An explanation of the survey's objectives, the amount of time required to complete it, and the guarantee that respondents would stay completely anonymous were provided at the outset. The decision of the potential participants to continue with the survey was taken as informed consent, and this was explicitly stated in the text.

### Statistical analysis

Continuous variables are presented as mean ± standard deviation as well as median value and interquartile range for non-normal distribution, while categorical data are presented as numbers and percentages. The intergroup differences were assessed using Student's t-test or Mann–Whitney test, where appropriate. In cases of 3 or more categories in the independent variable, one-way analysis of variance (ANOVA) was applied. Proportions were compared with chi-square test or Fisher's exact test when small expected numbers were detected. Statistical significance was considered at a level of *p* < 0.05. Statistical analysis was performed with the Statistical Package for the Social Sciences (SPSS) software, version 22.0 (IBM Corp., Armonk, NY, USA).

### Ethical considerations

The study was retrospectively reviewed and approved by the Ethics Committee in Research of the Medical University -Varna (№ 10/27.02.2025). The authors and co-authors declared that they have no conflicts of interest related to this article. No funding was used for the study, as it was conducted on an online platform in compliance with the criteria of the Helsinki declaration.

## Results

### Participant characteristics

There were 43 full-time, higher education medical staff members working in five PICUs in the country. The survey was completed by 37 (86%) respondents – two clinical psychologists and 35 physicians. More women than men took part in the study, as women represented 73% (*n* = 27) of the respondents. The medical staff's average age was 36.6 ± 7.6 years, from 26 to 56 years, median 34.5 (IQR 32.0–41.5) years. There was no significant age difference by sex. The majority of the physicians were between 30 and 40 years of age – 62.2% (*n* = 23) of all respondents. A high percentage (81%, *n* = 30) were married. More than half (51.4%, *n* = 19) thought that their job activities could interfere with their relationship, with about one third of their partners showing skepticism or discontent with their partner's work (29.7%, *n* = 11).

### Professional experience

**Figure 1:**
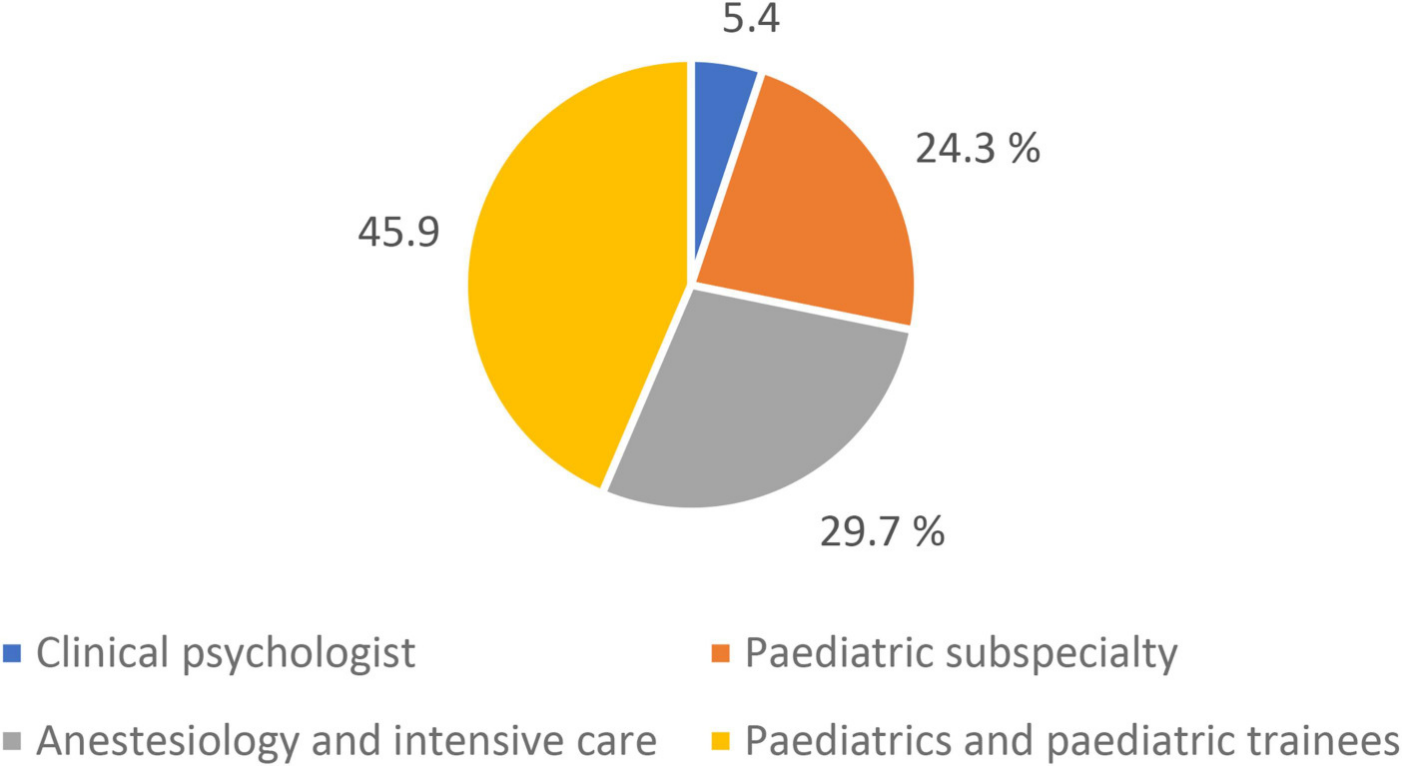
Distribution of the PICU healthcare staff according to medical specialty and subspecialty background.

Among respondents, 29.7% (*n* = 11) had first specialty in Anesthesiology and intensive care, while almost two thirds had or were currently training in pediatrics (70,2%, *n* = 26). Those with paediatric subspecialties (pediatric cardiology, pediatric neurology, pediatric nephrology and hemodialysis, neonatology, pediatric gastroenterology and toxicology) comprised 24.3% (*n* = 9). (Figure 1).

**Figure 2:**
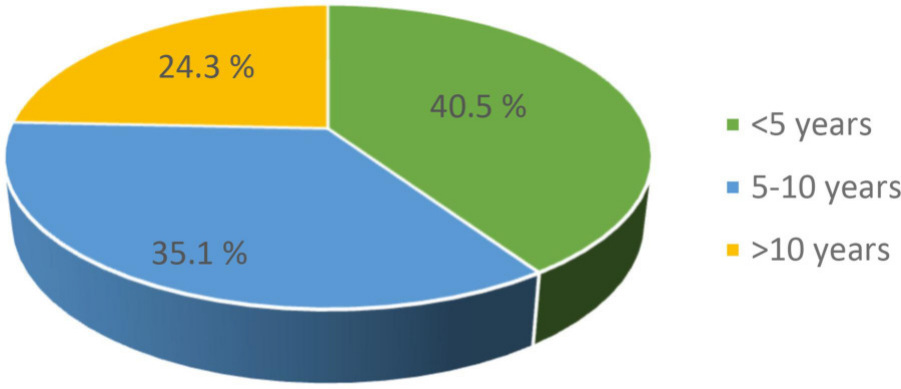
Distribution of PICU staff according to years of professional experience.

The majority of the participants had fewer than five years of work experience (Figure 2) while those with five to ten years of experience made up the next largest category.

### Working conditions

Among the respondents, 70.3% reported working 40–50 h per week, while 24.3% reported working more than 50 h per week. Working from home in their free time was reported by 42.5% (*n* = 15/35) of the respondents; 69.4% (*n* = 25/36) visited the workplace sometimes or often on weekends even when they were officially not at work, and 30,6% (*n* = 11/36) said they were not available outside working hours. No statistically significant association was observed between weekly working hours and burnout (*p* = 0.27), nor between work experience and burnout (*p* = 0.36); however, a tendency toward an association was noted, which did not reach statistical significance.

### Burnout and emotional burden

Among the staff with less than 5-year experience, 71.0% reported experiencing an episode of burnout, while 14.0% reported no such episode. Among those with > 10 years of experience, 55.6% reported having experienced burnout, whereas 33.0% reported no such episode. Nearly three-fourths of the respondents (73.0%, *n* = 27) described their workload as heavy but manageable. At the same time, although not significantly associated with burn-out, 32.4% (*n* = 12) of the participants reported frequently feeling overburdened by their work. More than half of the respondents reported occasionally feeling insecure in their work. In this context, “work process” refers to clinical decision – making task prioritization and the management of complex and critical situations. Among those reporting insecurity70.3% (*n* = 26) attributed this to work overload. Nevertheless, 73.0% (*n* = 27) of the respondents identified their work as a source of intellectual stimulation. A quarter of the respondents (27.0%, *n* = 10) were additionally burdened with institutional responsibilities. The respondents with a researcher's profile made up 63.9% (*n* = 23/36) and those with university teaching responsibilities – 56.8% (*n* = 21). Although 81% of the teachers reported burn-out compared to 60% of the non-academic staff, there was no statistically significant association between burnout and teaching (*p* = 0.479).

Over half of the physicians (54.1%, *n* = 20) occasionally considered leaving their current medical facility, and one out of seven often considered quitting. A total of 78.4% of respondents (*n* = 29) reported that higher monthly remuneration may reduce the likelihood of PICU physicians transitioning to the private sector.

**Figure 3:**
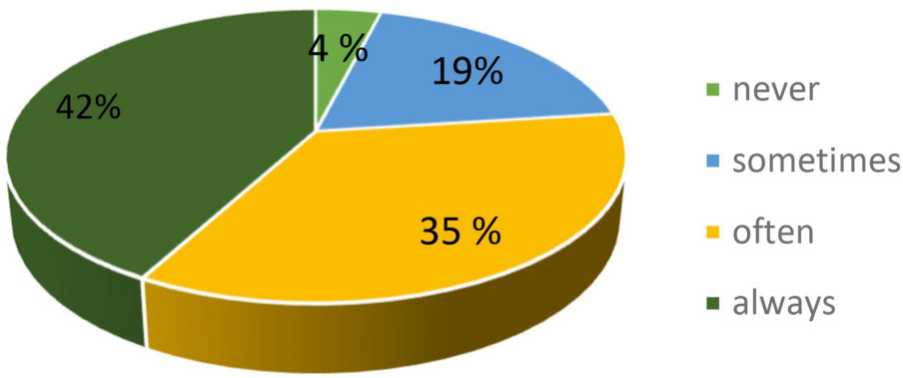
Distribution of the stress related to life-threatening situations among PICU staff members reporting at least one burnout episode.

After leaving their workplace, 62.2% (*n* = 23) of the respondents reported that they could not “break away” from work entirely; 75.7% (*n* = 28) coped moderately with the psychological burden of the patients they treated; 5.4% (*n* = 2) said they couldn't handle it at all. An episode of overheating, depression, and anxiety disorder was experienced by 73.0% (*n* = 27), and 75.6% (*n* = 28) reported sleep problems. The statistical analysis showed no significant association between burnout and sleep disturbances (Fisher's exact test, *p* = 0.41). More than once a week, 75.6% felt exhausted from work and 40.5% (*n* = 15) defined themselves as “crushed”. Only 2.7% (*n* = 1) reported no emotional burden while 70.2% (*n* = 26) reported experiencing emotional burden frequently and always in connection with stress of handling life-threatening situations. Looking at the distribution of staff experiencing stress in connection with life-threatening situations among those reporting burnout episodes (*n* = 27) revealed an interesting though not statistically significant, trend, *p* = 0.28 (Figure 3).

Emotional exhaustion was reported by 43.2% (*n* = 16) of all respondents; 64.8% (*n* = 24) were convinced that they worked too much at least once a week or more often; 37.8% (*n* = 14) used less than 25 days of annual leave; and 37.8% (*n* = 14) had to interrupt their annual leave at least once due to administrative reasons. There was no significant association between the interruption of holidays and burnout (*p* = 0.66). Over half of the respondents (51.4%, *n* = 19) reported feeling pressured occasionally while on vacation.

Overall, 51.4% of respondents (*n* = 19) reported being dissatisfied with their social life outside of medicine. Only 16.2% (*n* = 6) were fully satisfied with their monthly salary; 64.9% (*n* = 24) reported moderate satisfaction, whereas 18.9% (*n* = 7) indicated complete dissatisfaction. ANOVA didn't find any significant association between monthly remuneration and burnout (*p* = 0.442). The target salary was much higher among non-pediatric specialists – 73% chose the highest possible salary compared to 1/3 of pediatricians (*p* = 0.03). There was no significant correlation between the target salary and the specialists' age (*p* = 0.13). Work experience and target salary (*p* = 0.17), and work experience and monthly remuneration also didn't associate significantly (*p* = 0.265) Table 1.

**Table 1:** Associations between remuneration-related variables, professional characteristics, and burnout among PICU healthcare staff. Statistical significance was set at *p* < 0.05.

Variable	Comparison groups	*p*-value
Monthly remuneration	Burnout vs. No burnout	0.442
Monthly remuneration	Years of experience	0.265
Target salary	Pediatric vs. Non-pediatric	0.03
Target salary	Age groups	0.13
Target salary	Years of experience	0.17

### Professional satisfaction

Overall, 44.4% (*n* = 16/36) of the respondents reported satisfaction with their professional quality of life, and 56.8% (*n* = 21) had never considered changing the field in which they worked. Additionally, 70.3% (*n* = 26) reported that they would choose to work with children again; and 67.6% provided suggestions on how to improve the current situation. Young physicians with less than five years of experience desired more practical training, greater organization of their work, higher-quality and better structured training, adherence to well recognized clinical guidelines, and assistance from a specialist throughout their shifts. They also highlighted the need of better organization of the health care system, less bureaucracy, and improved remuneration; the opportunity for longer (at least 3 weeks) vacations twice a year; and an overall improvement of the working environment and equipment in the PICUs.

## Discussion

The current study shows for the first time quantitative data on work-related experience of Bulgarian doctors and psychologists, working in PICUs, provides sound evidence about substantial burnout, and suggests areas of improvement. In 1967, John Downes opened the first PICU in the United States at the Children's Hospital of Philadelphia (19). Intensive care services are now a crucial component of healthcare in developed nations (20). Although Paediatric intensive care (PIC) emerged as a new specialty in the field of medicine in the 1960s, with the awareness that a separate subspecialty is required to care for critically ill paediatric patients (21).

Healthcare professional burnout in itself is a global problem that negatively impacts staff productivity, patient safety, quality of patient care, retention and recruitment of medical staff (22, 23). One study in South Korea reported that there is an alarmingly high prevalence of burnout among intensivists and critical care fellows, with overall and severe burnout rates of 88.1% and 59.3%, respectively (24). It is therefore not unexpected that the majority of the workforce in the current study has worked in PICUs for less than five years, and that more than one in four doctors in PICUs has less than ten years of work experience. Burnout was more common among physicians than among their peers in the US, according to a survey of US medical students and residents (25). PICU staff are particularly at risk, with reported prevalence ranging from 40.0% to 70.0% (26–28). The present study reporting over 70.0% of employees experiencing distress burden, is not an exclusion. In terms of staff issues, it establishes the foundation for comparing the country's critical case medical care quality with that of other countries.

The working conditions and the specific activities, performed at PICUs can have important implications for practitioners’ mental health. In the present study, more than half of the physicians reported experiencing occasional insecurity in their work. This finding may reflect the complexity and high-stakes nature of clinical decision-making in this setting. Emerging technologies, including artificial intelligence (AI), have been proposed as potential tools to support clinical decision-making and reduce cognitive workload (29). Healthcare systems have to be flexible in their swift introduction. The younger PICU staff may be well positioned to adapt to such innovations (30). The current results demonstrate that PICU staff members already suffer from severe burnout and disrupted personal lives. We acknowledge that this may be worsened by the lack of structured PICU staff support in the country. Additionally, the study shows a higher percentage of female participants compared to male participants, which likely reflects the gender distribution of healthcare staff in PICU. Being the most numerous group in PICUs may be a sign that women act with responsibility and dedication in their professional life or, alternatively, it may just reflect the fact that Paediatrics in general is dominated by women (31). Historically, the sociological assignment of childcare as a woman's role made it also easier for female doctors to enter Paediatrics (32). The medical staff in this study is of young age, probably because this is the most attractive comprehensive specialty in medicine as it covers pathology from the neonatal period to the end of adolescence, a variety of diseases (infectious, surgical diseases, traumatology, and diseases of all organs and systems). The majority of the respondents emphasized the need for higher remuneration and improved working conditions (reduced total working time, longer yearly leave, less night shifts). All this would only be possible if Paediatric intensive care is recognized as a separate specialty in Bulgaria, similar to other countries. In 2020, a Turkish study examined the current situation of the Paediatric intensive care specialty and Paediatric intensive care units. It showed that there were 60 Paediatric intensive care units managed by PIC specialists (33). The current study showed that the majority of doctors in PICUs are paediatricians or other types of paediatric specialists. This is a reason to believe that with the recognition of the new specialty in PIC the leading physicians will have primarily paediatric background.

Despite the poor working conditions, the majority of the staff was dedicated and willing to work without complaining. In particular, measures such as increased remuneration, optimization of weekly workload, and improved shift organization may allow healthcare professionals sufficient time for recovery, family life, and personal commitments. In addition, the implementation of structured employee support programs and access to psychological counseling services may further mitigate burnout risk and enhance overall job satisfaction. Otherwise, over 16% of the respondents reported currently considering a job change, highlighting a potential risk for workforce attrition. This is consistent with results from other countries, such as 18% in Ireland in 2018 (34).

Professional dissatisfaction leads to a decline in the quality of patient care. Patients who are treated by doctors satisfied with their profession, receive a better quality of care (34, 35). It is important to mark that the respondents desire for higher salary was not tied to whether they were married, had children, their work experience, number of duties, gender or kind of specialty. Family obligations should not be neglected, as more than half of the respondents claimed that this could impede work and eventually cause overheating. Because of the detrimental effects of prolonged work in an intensive care unit, employees wanted their job to be adequately paid or compensated for. A decision of the European Court of Justice of 14 May 2019 sets an average working time for a seven-day workweek of 48 h, including overtime, for a typical 4-month reference period. More than two-thirds of the physicians who participated in the present survey reported working for 40–50 h per week, with almost a quarter reporting work exceeding 50 h per week. This could be compensated for by periodically assigning employees to wards with less rigorous work for a set amount of time or providing specializations in another city or country. Such an approach would stimulate both a “break” from work and an enrichment of knowledge in the field, which in turn would lead to an improvement in the quality of care in intensive care units.

According to the sample study, burnout rates among French physicians ranged from 28.0% to 73.0% across all disciplines (10). The number of night shifts was linked to an even higher rate of burnout in accordance with our study results (11). Employees working in non-university or private centers seem to be more satisfied with their work. Almost 2000 registered professionals were asked to rate the sources of satisfaction and dissatisfaction in New Zealand (36). Overall satisfaction was higher and dissatisfaction scores were lower in the private sector among 47.0% of the respondents. The respondents in our survey reacted negatively to administrative workload. This fact may have an additional negative impact on physicians, as clinical and scientific activities were conducted concurrently. Surprisingly, there was no correlation between teaching, research, and burnout. This highlights the well-known fact that creative work protects against burnout and justifies the place of PICUs in university hospitals (37).

To provide the necessary care for critically ill patients and their families, physicians must be in a favorable psychological state, satisfied with their work, and appreciated by their environment. As a result, the pediatric intensive care units could provide higher-quality care. Healthcare workers who experience psychological instability face a variety of personal and professional challenges, including decreased productivity, poor quality of care, absenteeism due to health or other reasons, use of psychoactive substances, smoking, alcohol use, interpersonal stress, as well as inferiority in family relationships. At our environment, a significant proportion of the personnel (around 70%) would still choose to work with children in spite of the challenges they encounter on a daily basis. This highlights the fact that such healthcare professionals work with love and passion which should be appropriately acknowledged. The recognition of PIC as a distinct board-certified specialty in Bulgaria separate from both pediatrics and adult critical care as it is recognized more than 40 years ago in other countries (38) may be associated with improvements in standards of care in PICUs and could potentially enhance the attractiveness of the field to younger physicians. Our study had **limitations**, since it was the first quantitative study on the burnout of Bulgarian doctors at PICUs as their workplace, and there was no basis for comparisons with previous data. Its cross-sectional design did not allow for conclusions of causality. The number of responses to this questionnaire was high for the short time of the study and representative for the overall work force at PICUs, but apparently small for performing a more meaningful quantitative analysis, as most of the trends did not reach statistical significance. A similar study is planned to be conducted among health care workers (nurses, midwives, etc.) working in PICUs as the emotional and psychological burden is likely to be comparable.

## Conclusion

Physicians employed in PICU deal with challenges on a daily basis that may affect negatively their emotional well-being and the quality of their work. Along with the responsibilities assigned to them, they must handle emergency situations, communicate daily with the relatives of the critically ill children, and often have to deliver bad news. The main reasons for dissatisfaction among respondents seem to be the excessive weekly working hours, administrative burden and the insufficient remuneration. Potential coping strategies include optimization of workload and shift organization, reduction of administrative burden, improved staffing levels, and the implementation of structured employee support programs, including access to psychological counseling services. Such measures may contribute to improved staff wellbeing, increased job satisfaction, and better retention of healthcare professionals. Despite the limitations, the present study findings contribute to an initial understanding of burnout among medical staff in Bulgarian PICUs and highlight the need for targeted interventions and further research to address this growing problem. Future efforts are needed to appreciate and acknowledge doctors for their work, to organize the PICUs in a better way in terms of manpower and recess in order to keep this important part of paediatric care intact and developing.

## Data Availability

The raw data supporting the conclusions of this article will be made available by the authors, without undue reservation.
