# Supplementary material for: Burnout among medical doctors working in paediatric intensive care units in Bulgaria
Source: Front Pediatr. 2026 Jun 1;14:1791485. doi: 10.3389/fped.2026.1791485 (PMC13265562; doi:10.3389/fped.2026.1791485)
Supplement: Supplementary file 1 [file Supplementaryfile1.docx]

Survey for study the Burnout syndrome among the medical staff working in Paediatric Intensive Care Units (PICUs) in Bulgaria

This survey is anonymous. The purpose of the study is to characterize the prevalence of burnout syndrome among medical professionals. It will take about 90 minutes to complete. The questionnaire adapted from the questionnaire dedicated for French neonatologist (Elodie Zana-Taïeb and team). We thank them for allowing us to adapt it. By continuing to the survey, you confirm your acceptance to participate. If you don’t want to take part, simply do not continue beyond this point.

1. What is your gender?

- Male
- Female

1. How old are you? (in completed years)
2. What is your specialty?
3. How long have you been working in PICU?
   - <5 years
   - <10 years
   - >10 years
4. In which region of the country do you work?
   - Northeast Bulgaria
   - Northwest Bulgaria
   - Southeastern Bulgaria
   - Southwest Bulgaria
   - Central Bulgaria
5. In which city do you work?
6. What is the position in PICU do you hold?
7. Do you work on a permanent employment contract?
8. What is the status of the hospital where you are working?
   - University State Hospital
   - University Private Hospital
   - Non-university public hospital
   - Another kind of hospital
9. If you work in a hospital, do you currently have a private practice outside of working hours?
   - Yes
   - No
10. How many beds do you have in the PICU?
    - <5
    - 5-10
    - >10
11. Have you ever felt insecure in the medical facility where you perform your main activity?
    - Never
    - Rarely
    - Sometimes
    - Often
    - Always
12. If yes, for what reason(s)?
    - Insufficient technical support
    - Team cohesion problem
    - Work overload
    - Other - free answer
13. Do you feel overwhelmed by the activity in the medical facility where you work?
    - Never
    - Rarely
    - Sometimes
    - Often
    - Always
14. Do you have institutional responsibilities (head of ward, head of department, chairman of an institutional medical committee, participant in an institutional medical committee)?
    - Yes
    - No
15. Would you like to apply for such a position?
    - Yes
    - No
16. Are you a teacher?
    - Yes
    - No
17. Have you got a research activity ?
    - Yes
    - No
18. Do you work full time or part time?
    - Full time
    - Part-time
19. How many hours per week do you work at your workplace?
    - < 40 hours / week
    - 40-50 hours / week
    - 50-60 hours / week
    - >60 hours / week
20. How many half-days per week do you work at the hospital/clinic outside of on-call duty?
    - < or equal to 2
    - 3-4
    - > or equal to 5
21. How many half-days per week do you attend consultations (of any type)?
    - < or equal to 2
    - 3-4
    - > or equal to 5
22. Do you have shifts (duties)?
    - Yes
    - No
23. How are your shifts organised?
    - On-site duty
    - On-call security duty
    - On-site duty + security duty
24. What is the duration of your on-call duty?
    - 6 h
    - 8 h
    - 12 h
    - Other
25. How many people are included in the duty schedule?
    - <5
    - 6-8
    - 9-11
    - >12
26. How many people are involved in the on-call schedule?
    - 0
    - 1
    - 2
    - Other
27. On average, how many shifts do you have per month?
    - <5
    - 6-8
    - 9-12
    - >12
28. On average, how many weekends per month are you on call?
    - <=1
    - 2-3
    - 4
    - I have no calls on weekends
29. Do you take breaks at work?
    - Yes, always
    - Yes, sometimes
    - No, never
30. If yes: what do you mostly do on your on-call rest? (maximum 3 answers)
    - Sleeping
    - Free time
    - Family time
    - Household chores
    - Administrative / professional
    - Other (hobbies, scientific works, etc.)
31. Do you think that childcare has a negative impact on your personal life (fatigue, anxiety, burden on loved ones, etc.)?
    - Strongly disagree
    - Moderately agree
    - Totally agree
32. If you had a choice, would you give up your on-call activity?
    - No, but I would limit it
    - Yes, I would
    - No, I wouldn't
33. What is the work in the department/clinic like for you?
    - A source of intellectual stimulation
    - Financial interest
    - Just a work
    - Other
34. Do you think your working hours are limiting?
    - Completely
    - Moderately
    - Not at all
35. Do you work at home in the evening?
    - Never
    - Rarely
    - Often
36. Do you go to your workplace at the weekend, even if you are not on call?
    - Never
    - Sometimes
    - Often
37. Are you available off-duty?

- Never
- Sometimes
- Often
- Always

1. How would you rate your workload?
   - Light
   - Moderate
   - Heavy and manageable
   - Heavy and difficult to manage
2. During your work, have you thought about the legal aspects of your work in an intensive care unit?
   - Never
   - Sometimes
   - Often
   - Always
3. Have you ever been tried (conciliation, expertise, justice)?
   - Yes
   - No
   - I have been investigated, but not tried
   - I have been questioned in court cases
4. If yes, on a scale of 0 to 10, how much did the situation affect you? (free answer)

0 - It didn't affect me at all, 10 - it affected me a lot

1. Do you feel recognized by those around you?
   - Completely
   - Moderately
   - Not at all
   - I haven't thought about it
2. Did you have a person during your studies who influenced your career choice?
   - Yes
   - No
3. Did you experience discrimination in your profession because of your gender?
   - Yes
   - No
4. Do you think your career choice would have been different if you were of the opposite sex?
   - Yes
   - No
5. Do you feel worried about the difficulties facing the profession (staff burnout, lack of recognition from politicians, etc.)?
   - I'm not worried at all
   - I am moderately worried
   - I am totally worried
6. Have you ever considered leaving the hospital to work as a specialist in private sector?
   - Never
   - Sometimes
   - Often
7. Paediatricians and their orientation in the private sector: how to keep the public sector attractive? (multiple answers are possible)
   - Increase in basic wages
   - Increase in monthly cash supplements
   - Call time pricing
   - An additional half day off
   - Other
8. Does it cause you stress when you have to deal with a life-threatening emergency?
   - Never
   - Rare
   - Sometimes
   - Often
   - Always
9. Are you able to "break away" when you leave the hospital/clinic?
   - Easy
   - Moderately
   - Hard
10. Are you able to cope with the psychological burden of the cases you treat?
    - Absolutely yes
    - Moderately yes
    - Not at all
11. If no, have you sought outside help for this?
    - Yes
    - No
    - I've thought about it, but I don't know where to find such help
12. Have you ever had an episode(s) of burnout/depression? (Related to your work)
    - Yes
    - No
    - I am not sure
13. Do you have sleeping problems?
    - Yes
    - No
    - Sometimes
14. Do you smoke tobacco?
    - Yes
    - No, I don't
    - Occasionally
15. How often do you drink alcohol?
    - Never
    - <1 time / month
    - < 2 times / month
    - About once a week
    - Several times a week
    - Everyday
16. Have you ever used other psychoactive substances?
    - Yes
    - No
17. If yes, which ones? Optional answer
    - Cannabis
    - Cocaine
    - Neuroleptic
    - Other
18. Do you ever feel emotionally drained by your work?
    - Never
    - Sometimes a year
    - Once a month
    - More than once a month
    - Once a week
    - Sometimes a week
    - Every day
19. Do you ever feel exhausted at the end of your working day?
    - Never
    - Sometimes a year
    - Once a month
    - More than once a month
    - Once a week
    - Sometimes a week
    - Every day
20. Do you ever feel like you are cracking up because of your work?
    - Never
    - Sometimes a year
    - Once a month
    - More than once a month
    - Once a week
    - Sometimes a week
    - Every day
21. Do you ever feel frustrated by your work?
    - Never
    - Sometimes a year
    - Once a month
    - More than once a month
    - Once a week
    - Sometimes a week
    - Every day
22. Do you sometimes think that you work "too hard"?
    - Never
    - Sometimes a year
    - Once a month
    - More than once a month
    - Once a week
    - Sometimes a week
    - Every day
23. What is your current personal situation?
    - Married/ in a permanent relationship
    - Single
24. Do you feel that your profession can be an obstacle in your relationship?
    - Yes
    - No
25. Is your spouse a support in your career choice?
    - Yes
    - No
26. What is your spouse's profession?
    - Farmer/operator
    - Craftsman
    - Trader
    - Company manager
    - Executive
    - Employee
    - Teacher
    - Engineer
    - Doctor / surgeon
    - Legal professions
    - Worker
    - Paramedical profession
    - Pharmacist
    - Unemployed
    - Other
27. Which member of the couple brings in the largest salary?
    - You
    - Your spouse
28. Do you have children?
    - 0
    - 1
    - 2
    - 3
    - >3
29. How do you rate your parent-child relationship(s)?
    - Completely satisfactory
    - Moderately satisfactory
    - Not at all satisfactory
30. Who does the housework in your home? (Multiple answers possible)
    - You
    - Your spouse
    - Chores shared equally with your spouse
    - Domestic worker
    - Domestic worker & joint tasks
31. Do you play sports?
    - Yes
    - No
    - I would like to, but I don't have time
32. If yes, how often?
    - < 1 time per month
    - > 1 time per month
    - > 1 time per week
    - <1 time per week
33. Do you engage in any extra-professional/artistic activities other than sport?
    - Yes
    - No
34. How many days of holiday do you take per year?
    - < 20 days per year
    - 20-25 days per year
    - 25-30 days per year
    - > 30 days per year
35. Have you ever had your leave interrupted for administrative work-related reasons?
    - Yes
    - No
36. Do you sometimes feel under pressure while on vacation? (In relation to your occupation)
    - Never
    - Sometimes
    - Often
37. Do you think you have enough time to devote to your well-being?
    - Completely
    - Moderately
    - Not at all
38. How do you see your social life outside of medicine?
    - Very satisfactory
    - Moderately satisfactory
    - I am not satisfied at all
39. Are you satisfied with your remuneration?
    - I am completely satisfied
    - Moderately satisfied
    - I am not satisfied at all
40. What is your monthly salary?
    - < 750 Euro/ month
    - 750-1000 Euro/ month
    - 1000-1250 Euro/ month
    - 1250-1500 Euro/ month
    - 1500 – 1750 Euro/ month
    - 1750-2000 Euro/ month
    - > 2000 Euro month
41. What would be your "target" salary?
    - 1000-1250 Euro/ month
    - 1250-1750 Euro/ month
    - 1750 – 2250 Euro/ month
    - 2250-2500 Euro/ month
    - > 5000 Euro/mont
42. Are you satisfied with the pay for the shifts?
    - I am completely satisfied
    - Moderately satisfied
    - I am not satisfied at all
    - I do not receive a separate remuneration for being on duty
43. What is the value of your duty?
    - Between 25-50 Euro
    - Between 50-75 Euro
    - Between 75-100 Euro
    - Between 100-125 Euro
    - > 125 Euro
44. Are you satisfied with the quality of your professional life?
    - Yes
    - No
    - I don’t know
45. On a scale of 0 to 10, how would you rate the quality of your work life? (free answer) 0-very bad; 10 – excellent
46. Have you ever thought of changing the field in which you work?
    - Often
    - Sometimes
    - Never
47. If you had to start over, would you choose to be paediatrician again?
    - Yes
    - No
    - I don’t know
48. If not, which specialty would you choose? (free answer)
49. If not, which criterion would be most important when choosing another specialty?
    - better pay
    - no on-call or weekend work
    - more satisfactory working hours
    - flexibility of planning
50. As an intensivist, if you had it to do over again, would you make the same medical career choice?
    - Yes
    - No
    - I don’t know
51. What changes would allow you to have a better quality of life? (free answer)
